# A telomerase-associated RecQ protein-like helicase resolves telomeric G-quadruplex structures during replication

**DOI:** 10.1016/j.gene.2012.01.068

**Published:** 2012-04-15

**Authors:** Jan Postberg, Maksym Tsytlonok, Daniela Sparvoli, Daniela Rhodes, Hans J. Lipps

**Affiliations:** a Witten/Herdecke University, Centre for Biomedical Education and Research (ZBAF), Institute of Cell Biology, Witten, Germany; b Medical Research Council Laboratory of Molecular Biology, Cambridge, UK; c HELIOS Medical Centre Wuppertal, Department of Paediatrics, Wuppertal, Germany

**Keywords:** TEBP, telomere-binding-protein, RNAi, ribonucleic acid interference, *E. coli*, *Escherichia coli*, DNA, deoxyribonucleic acid, RNA, ribonucleic acid, TSP, telomere-suppression PCR, TERT, telomerase reverse transcriptase, TR, telomerase RNA subunit, SDS, sodium dodecyl sulphate, PAGE, polyacrylamide-gel electrophoresis, UTR, untranslated region, ssDNA, single-stranded DNA, dsRNA, double-stranded RNA, Macronucleus, Replication band, Tert, *Stylonychia lemnae*, Ciliates

## Abstract

It is well established that G-quadruplex DNA structures form at ciliate telomeres and their formation throughout the cell-cycle by telomere-end-binding proteins (TEBPs) has been analyzed. During replication telomeric G-quadruplex structure has to be resolved to allow telomere replication by telomerase. It was shown that both phosphorylation of TEBPβ and binding of telomerase are prerequisites for this process, but probably not sufficient to unfold G-quadruplex structure in timely manner to allow replication to proceed. Here we describe a RecQ-like helicase required for unfolding of G-quadruplex structures in vivo. This helicase is highly reminiscent of human RecQ protein-like 4 helicase as well as other RecQ-like helicase found in various eukaryotes and *E. coli*. In situ analyses combined with specific silencing of either the telomerase or the helicase by RNAi and co-immunoprecipitation experiments demonstrate that this helicase is associated with telomerase during replication and becomes recruited to telomeres by this enzyme. In vitro assays showed that a nuclear extract prepared from cells in S-phase containing both the telomerase as well as the helicase resolves telomeric G-quadruplex structure. This finding can be incorporated into a mechanistic model about the replication of telomeric G-quadruplex structures during the cell cycle.

## Highlights

*► Stylonychia* StyRecQL is a helicase reminiscent of human RecQ protein-like 4. ► StyRecQL is enriched in replication band of the macronucleus during S-phase. ► StyRecQL colocalizes and is associated with Tert during telomere replication.

## 1 Introduction

Telomeres, the DNA-protein complex at the termini of eukaryotic chromosomes are essential for genome integrity (Rhodes and Giraldo, 1995). Telomeric DNA consists of simple tandem arrays of repeated sequences in which the 3′-strand is very guanine-rich and forms a 3′ single stranded overhang. The length of telomeric duplex DNA varies between organisms, from 20 bp in some ciliated protozoa up to over 10 kbp in mammals. The 3′-overhang is essential for telomere function and due to its high concentration of guanines can form stable G-quadruplex structures (for review: Maizels, 2006; Lipps and Rhodes, 2009). Telomeres are associated with proteins, both binding to the duplex and single-stranded telomeric sequences. Although the composition of this protein complex varies from species to species some of these proteins are highly conserved from yeast to human (Linger and Price, 2009). For example the yeast Cdc13 and TEBPα from ciliates both bind to the single-stranded 3′-overhang and are homologous both in structure and function to the human POT1 (Baumann and Cech, 2001). More recently it was found that the human TPP1 is the human homolog to the ciliate TEBPβ and both are involved in telomerase recruitment (Paeschke et al., 2008b; Wang et al., 2007).

It is believed that in addition to telomere-associated proteins higher order DNA structures are crucial for telomere function. One of these structures is the T-loop found by electron microscopy in a number of species, Trypanosomes, yeasts, ciliates, nematodes and mammals (de Lange, 2004; Griffith et al., 1999). In this structure the single stranded overhang invades the double stranded telomeric region of the same chromosome. It is not known whether T-loops are formed at each telomere, how they are regulated during the cell cycle or how they are resolved. The other secondary structure that can affect telomere function are G-quadruplexes in which four guanines associate into a cyclic Hoogsten hydrogen bonding arrangement in the presence of monovalent ions (Burge et al., 2006; Rhodes and Giraldo, 1995). G-quadruplex DNA structures are highly polymorphic (Patel et al., 2007) but the formation of the intermolecular antiparallel G-quadruplex structure at the telomeres of the spirotichous ciliate *Stylonychia* has been demonstrated to occur *in vivo* (Schaffitzel et al., 2001). Since the macronucleus of this species contains 10^8^ telomeres, telomeric G-quadruplex structures could be visualized by using single-chain antibodies directed against the antiparallel intermolecular G-quadruplex structure. Moreover, since replication of macronuclear DNA occurs in a morphological distinct region, the replication band, it could be shown that telomeric G-quadruplex structure becomes resolved during replication. The loss of telomeric DNA during replication due to the end-replication problem (Vega et al., 2003) is prevented by a specialized enzyme, the telomerase, which uses its RNA component to template extension of the 3′-end while the complementary strand can be synthesized by conventional RNA-primed DNA replication (Gilson and Geli, 2007). It has been shown before *in vitro* that telomeric G-quadruplex structure prevents the action of telomerase in *Oxytricha* and other species although this may not hold true for all organisms (Oganesian et al., 2006; Wang et al., 2011; Zahler et al., 1991; Zhang et al., 2010).

The regulation of G-quadruplex structure during the cell cycle has been extensively studied in the ciliate *Stylonychia* using antibodies specifically recognizing G-quadruplex DNA (Paeschke et al., 2005, 2008a, 2008b; Schaffitzel et al., 2001). Here it was shown that the C-terminus of TEBPβ is responsible for the folding of the telomere into G-quadruplex structure and that both, phosphorylation of TEBPβ and binding of telomerase to the telomeres during replication are necessary prerequisites for unfolding of this structure during replication. These experiments could not distinguish whether binding of telomerase accelerates G-quadruplex unfolding during replication or whether a telomerase-associated G-quadruplex-specific helicase might be actively involved in this process.

G-quadruplex DNA structures are much more stable than double-stranded DNA and a variety of helicases, such as for example RecQ, Pif1, FANC-J, have been shown to unfold G-quadruplex structures *in vitro.* Furthermore loss of function of some of these helicases leads to genome instability syndroms (reviewed in Huber et al., 2006; Lipps and Rhodes, 2009; Maizels, 2006; Paeschke et al., 2010). In this report we describe the identification of a telomerase-associated helicase required for the *in vivo* unfolding of telomeric G-quadruplex structure during replication.

## 2 Material and methods

### 2.1 Characterization of a putative RecQ-like helicase in *Stylonychia lemnae*

Growth of *Stylonychia* cells, isolation of macronuclear DNA and synchronization of cells was performed as described before (Ammermann et al., 1974; Juranek et al., 2000). To identify putative conserved proteins similar to G-qudruplex-binding helicases we designed degenerate primers targeted to the conserved regions of the central helicase domains of various eukaryotic helicases (P1_degenerate_: GAY TAR GCH CAY TGY GTN TC; P2_degenerate_: GDA TNA CRA ANC GNA CRT C). Using these primers for PCR we obtained a 570 bp DNA fragment encoding a RecQ-like helicase (StyRecQL). Subsequently we used this sequence information to design *Stylonychia*-specific primers for the characterization of the whole nanochromosome by telomere suppression PCR (TSP), a technique to amplify the 5′- or 3′-ends of *Stylonychia* nanochromosomes including their telomeric sequences (Curtis and Landweber, 1999), eventually resulting in a complete nanochromosomal sequence.

Total RNA was isolated from proliferating vegetative cells by guanidinium thiocyanate-phenol-chloroform extraction using Trizol reagent (Invitrogen). We then stepwise determined the mRNA sequence of StyRecQL from cDNA obtained from macronuclear RNA by reverse transcription (Qiagen One Step RT-PCR kit). A primer walking strategy was used starting at the central conserved domain. The resulting overlapping amplicons were TA-cloned into pGEM-T easy vector (Promega), sequenced and used for the reconstruction of the mRNA sequence.

### 2.2 Silencing of StyRecQL gene expression

**Fig. 1:**
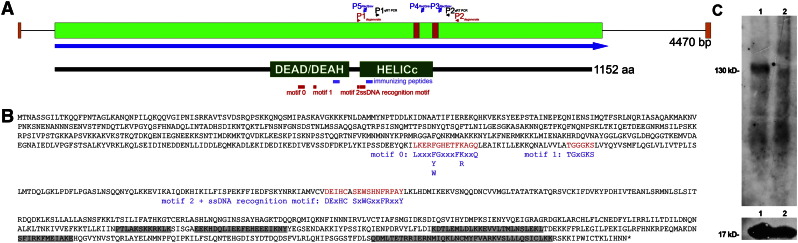
*Gene structure and conserved regions of the putative RecQ-like helicase.* StyRecQL is encoded on a 4470 bp nanochromosome. A. The cartoon illustrates the genomic organization of StyRecQL. The positions of a degenerate primer pair (red arrows), gene-specific primers used for telomere-suppression PCR (blue arrows) and qRT PCR primers are indicated on top. The nanochromosome is flanked by telomeres at both ends (orange) and possesses a ~ 220 bp 5′-subtelomeric region. Since we only partially characterized the mRNA sequence (blue), the transcription start site is not known. However, the protein coding sequence (light green) is interrupted by two short CT-AG introns (red), followed by a ~ 700 bp 3′-subtelomeric region. The predicted protein coding sequence encodes an 1152 aa protein with a conserved central helicase domain (dark green), which commonly habours several motifs conserved StyRecQL. Predicted α-helices in the C-terminus of StyRecQL are shaded gray. The positions of several motifs as well as peptides used for immunization of rabbits are indicated. B. Protein sequence of StyRecQL. The exact positions of several conserved motifs and consensus sequences are given. C. Western analyses demonstrate the occurrence of StyRecQL in macronuclear extract as an approximately 130 kDa protein (lane 1) as well as its knock down upon RNAi treatment (lane 2). As a loading control we used the same samples and probed them with antibodies directed against histone H3 (~ 17 kDa) (below lanes 1 and 2).

Silencing of TERT gene expression by RNAi followed the protocol of (Paeschke et al., 2008b). Silencing of StyRecQL was performed using sequence motifs encoding the whole central conserved domain of StyRecQL as a target. To raise antibodies specific for the putative RecQ homolog two peptides from the helicase domain unique for StyRecQL, as determined by tBlastn homology searches in *Stylonychia* and, since the macronuclear genome data of this ciliate is still incomplete, the closely related *Oxytricha*, were chosen for immunization of rabbits by co-injection (peptide 1: CGASLNSQQNYQLKK; peptide 2: CLHDMIKEKVSNQQD). Specificity tests revealed that the antisera recognized an ~ 130 kDa band in Western analyses using macronuclear extracts (compare Fig. 1C, lane 1), whereas this band could not be detected in a peptide competition assay — here, the immunizing peptides where incubated in excess with the anti-StyRecQL antisera prior to incubation in Western analyses (data not shown). Antibodies directed against the *Stylonychia* telomerase protein raised in chicken were used as described earlier (Paeschke et al., 2008b).

### 2.3 In situ analyses

*In situ* antibody staining was done as described before (Postberg et al., 2008; Schaffitzel et al., 2001, 2010) using a Zeiss LSM 5 Pascal confocal microscope, co-immunoprecipitation and Western analyses were either performed from a telomerase extract (Froelich-Ammon et al., 1998) or from a nuclear extract following the protocols described by (Heyse et al., 2009; Postberg et al., 2008). At least 5 to 10 nuclei were inspected for each experiment.

### 2.4 In vitro analyses

For the *in vitro* helicase assay a model telomere (Paeschke et al., 2005) was incubated in 0.5 M NaCl overnight. The helicase assay was made in 10 mM Tris 7.2, 5 mM CoCl_2_, 5 mM ATP, 100 μg BSA/ml and 400 μg/ml sheared salmon sperm DNA. 0.5 nM model telomere and 10 μl telomerase extract was used per reaction and performed at 37 °C for various time intervals. As negative control a telomerase extract was used to which 1% SDS was added and heated for 10 min at 65 °C for protein denaturation. Telomeric DNA was separated by 10% PAGE (Sundquist and Klug, 1989).

## 3 Results

### 3.1 Characterization of a putative RecQ-like helicase in the macronuclear genome

Using degenerate primers targeted to the conserved regions of the central helicase domains of various eukaryotic RecQ helicases we could amplify a 570 bp DNA fragment from macronuclear DNA. Sequence analyses revealed that this fragment could derive from a nanochromosome encoding a RecQ-like helicase. Subsequently we used this sequence information to design *Stylonychia*-specific primers for the characterization of the whole nanochromosome by TSP as described in Material and methods (Curtis and Landweber, 1999). Eventually we were able to amplify and characterize a complete 4.462 bp nanochromosome encoding a putative RecQ protein-like helicase (StyRecQL) in this ciliate (GenBank accession: JN377735). Similarity searches against raw sequence fragments of a *Stylonychia* macronuclear genome database, which was obtained by high throughput sequencing using the Roche 454 platform, suggested that to our present knowledge StyRecQL could be the only RecQ protein-like helicase found in *Stylonychia*. StyRecQL encodes a predicted 1152 aa protein with an estimated size of 131.33 kDa and contains 2 GT-AG-introns (34 bp or 35 bp, respectively) downstream of the region encoding the central conserved DEAD/DEAH and HELICc domains as determined by comparing the genomic sequence and the mRNA. The gene is flanked by a 237 bp 5′-UTR and a 702 bp 3′-UTR. A schematic diagram of the macronuclear nanochromosome encoding this helicase is shown in Fig. 1. To strengthen the hypothesis that a RecQ protein-like helicase is encoded by the 4.462 bp nanochromosome we compared the translated protein sequence with RecQ-like helicase sequences already characterized in other eukaryotes and *E. coli*.

The RecQ helicases Sgs1p and BLM in *S. cerevisiae* have been shown to unwind G-quadruplex DNA preferentially in comparison to Holliday junctions (Huber et al., 2002). By deletion mutant experiments the recognition of G-quadruplex DNA has been mapped to the central helicase domain of Sgs1p. *E. coli* RecQ is only 609 aa in length. In contrast eukaryotic RecQ helicases are frequently more than twice as large, possibly reflecting the presence of additional functional domains or motifs for protein interactions. Eukaryotic helicases share a central domain with a length of approximately 400 aa, which commonly includes the catalytically active N-terminal helicase and RecQ-C-terminal (RQC) subdomains as well as the substrate binding C-terminal HRDC domain (*helicase-and-RNase-D C-terminal*). The N-terminal helicase domain of RecQ helicases usually habours several conserved motifs we also identified in StyRecQL, which are most probably involved in ATP binding and hydrolysis (motif 0, motif 1, motif 2) as well as ssDNA recognition (Table 1) (Bennett and Keck, 2004).

In most bacterial and eukaryotic RecQ proteins characterized to date the helicase domain is followed by a conserved RQC subdomain, whose Zn^2+^ binding region apparently plays an important role in RecQ associated functions, such as DNA, or possibly G4 binding. Remarkaby, human RecQ protein-like 4 and alternatively spliced isoforms of the human RECQ5 gene lack this region. It has been shown in *S. cerevisiae* that Zn^2+^ has an inhibitory effect of Sgs1p-mediated DNA unwinding, suggesting that Zn^2+^-binding sites in eukaryotic RecQ helicases may have negative regulatory function. Similarly as in human RecQ protein-like 4, we could neither identify a conserved RQC domain in StyRecQL nor cystein arrays, which are frequently found in RecQ homologs of *E. coli* and eukaryotes. Experimental data from numerous studies suggest that the C-terminal HRDC domains from various RecQ helicases, which contain multiple α-helices, have at least auxiliary function in DNA-binding (including ssDNA, as suggested from experiments in yeast. In detail the HRDC domain of Sgs1p preferentially binds ssDNA (Bernstein and Keck, 2005; Liu et al., 1999), whereby this domain is structurally less conserved when compared to other domains in RecQ helicases. The C-terminal domain of StyRecQL is predicted to contain 5 α-helices (gray shaded in Fig. 1B) using the NetSurfP algorithm for secondary structure prediction (Petersen et al., 2009). However, by sequence similarity searches and domain analyses using Prosite (Sigrist et al., 2010) we were unable to identify a conserved HRDC domain. In summary, the StyRecQL helicase identified in *Stylonychia* exhibits a domain organization reminiscent of human RecQ protein-like 4 with a central helicase domain including several sequence motifs characteristic for RecQ-like helicases (motifs 0–2 and ssDNA recognition motif), but no RQC or HRDC domain could be identified.

**Figure A.1:**
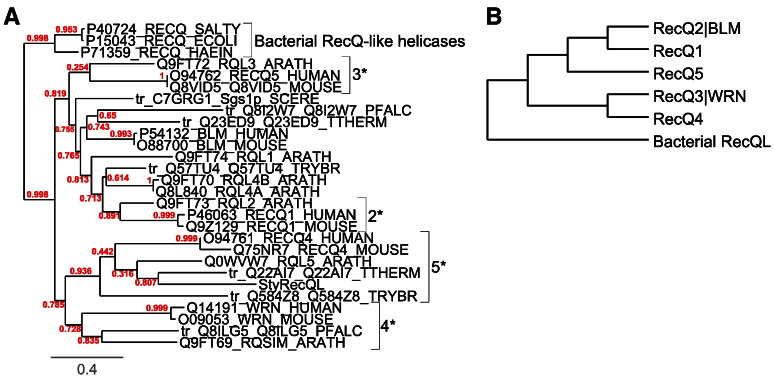
*Phylogenetic analysis of 28 RecQ protein sequences from various eukaryotic groups and bacteria.* A. The evolutionary history was inferred by estimating maximum likelihood phylogenies (Dereeper et al., 2008; Guindon et al., 2010) from the same RecQ-like protein sequences used above, which were realigned with MUSCLE (Edgar, 2004). Branch support values (red) are positioned next to the corresponding branches. The clade containing the bacterial RecQ-like proteins was used to root the tree. Notably, the topology of the resulting tree is very similar to the tree obtained when using the Neighbor-Joining method (compare Fig. 2). In detail, the cladistic relationship between clades 2*, 3*, 4* and 5* correlates between both analyses, whereas clade 1* (compare Fig. 2*) was not supported. B. The evolutionary history of the RecQL family of helicases was derived from phylogenetic analyses using the Neighbor-joing method or a Maximum Likelihood algorithm as described. Importantly, homologous RecQL proteins seem to occur in distantly related eukaryotes suggesting an old origin of all RecQL family members.

**Fig. 2:**
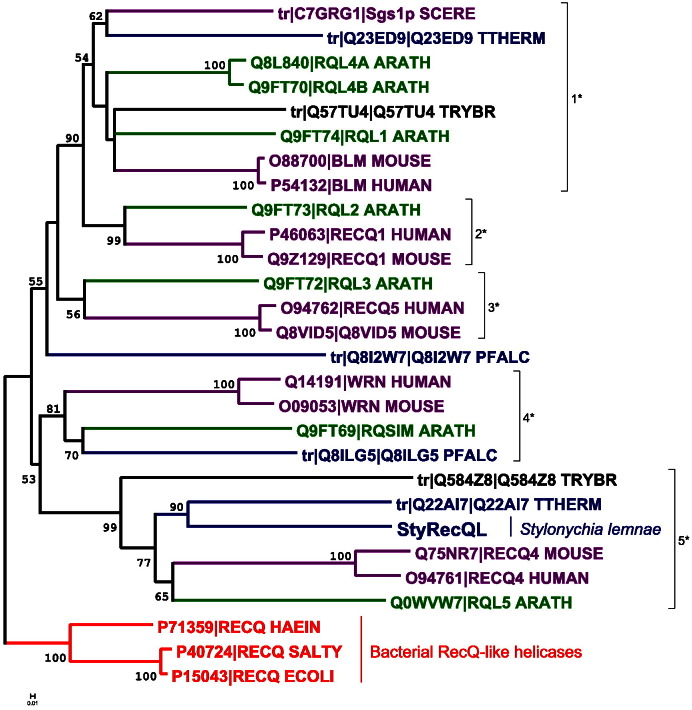
*Phylogenetic analysis of 28 RecQ protein sequences from various eukaryotic groups and bacteria.* The evolutionary history was inferred using the Neighbor-Joining method (Saitou and Nei, 1987). The bootstrap consensus tree inferred from 2000 replicates is taken to represent the evolutionary history of the taxa analyzed (Felsenstein, 1985). Bootstrap values > 50 are shown. Evolutionary distances were computed using the JTT matrix-based method (Jones et al., 1992) and are in the units of the number of amino acid substitutions per site. All positions containing gaps and missing data were eliminated from the dataset (Complete deletion option). There were a total of 311 positions in the final dataset. Phylogenetic analyses were conducted in MEGA4 (Tamura et al., 2007). The bacterial clade was used as outgroup to root the tree. Various clades contain RecQ-like proteins from distantly related eukaryotic groups (Parfrey et al., 2011), such as clade 1* (opisthokonts/purple, alveolates/blue, plants/green, excavates/black), clades 2* and 3* (opisthokonts/purple, plants/green), clade 4* õ(opisthokonts/purple, alveolates/blue, plants/green) as well as clade 5* (opisthokonts/purple, alveolates/blue, plants/green, excavates/black). Abbreviations used: ECOLI (*Escherichia coli*), HAEIN (*Haemophilus influenzae*), SALTY (*Salmonella typhimurium*), ARATH (*Arabidopsis thaliana*), TRYBR (*Trypanosoma brucei*), TTHERM (*Tetrahymena thermophila*), PFALC (*Plasmodium falciparum*), SCERE (*Saccharomyces cerevisiae*).

Phylogenetic analysis of 28 RecQ protein sequences from various eukaryotic groups and bacteria revealed that various clades contain RecQ-like proteins from distantly related eukaryotic groups, which are well supported by recent deep phylogenetic analyses of the eukaryotic evolutionary history (Parfrey et al., 2011). Our analyses using alternative algorithms to infer phylogenetic trees suggest that diversification of RecQ-like proteins occurred early in evolutionary history (Fig. 2, Fig. A.1A,B). Moreover, together with RecQ-like proteins from Trypanosoma (excavates) and Arabidopsis (plants), StyRecQL as well as a putative RecQ-like protein from another ciliate (*Tetrahymena thermophila*) reside within the same clade as mammalian RecQ4-like proteins. We therefore assume that StyRecQL could be a RecQ protein-like helicase homologous to human RecQ protein-like 4.

### 3.2 Functional analyses

To elucidate the possible function of this putative helicase, *in situ* analyses and co-immunoprecipitation experiments were performed. To obtain specific antibodies rabbits were immunized with peptides specific for StyRecQL (Fig. 1B). Subsequently the expression of either telomerase or the helicase was silenced by RNAi and the effect on G-quadruplex structure during replication, as well as the behaviour of telomerase and the helicase after silencing were analyzed. Finally, an attempt was made to demonstrate an *in vitro* G-quadruplex unfolding activity of this helicase.

As shown in lane 1 in Fig. 1C the anti-StyRecQL antibody reacts in a Western blot analysis using macronuclear extracts with a single protein of approximately 130 kD. Notably, when *Stylonychia* cells were RNAi-treated prior to Western analyses using dsRNA specific to StyRecQL no protein could be detected using anti-StyRecQL antibodies (Fig. 1C, lane 2).

**Fig. 3:**
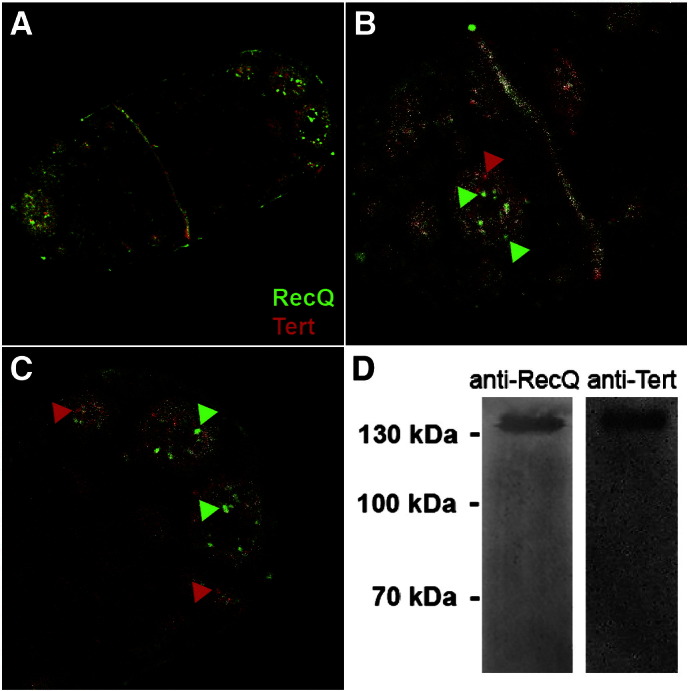
*Immunofluorescence analyses of StyRecQL and TERT localization within the macronucleus.* A–C. Immunofluorescence analyses exhibit the macronuclear localization of StyRecQL (green) as well as TERT (red). B–C. Colocalizing voxels are colored in white, whereas non-colocalizing voxels occur as green (StyRecQL) or red (TERT) signals. Green or red arrows point to selected examples of non-colocalizing voxels. D. Western analyses of co-immunoprecipitation experiments using the anti-StyRecQL antibody (left lane) or anti-TERT antibody (right lane) for detection.

Immunofluorescence analyses using anti-StyRecQL antibodies showed that StyRecQL occurred as foci-like accumulations within multiple spherical chromatin-poor nuclear bodies in macronuclei during interphase. Furthermore during S-phase StyRecQL was enriched in the replication band, while the nuclear distribution of StyRecQL outside of the replication band was reminiscent of interphase macronuclei (Figs. 3A,B,C).

In earlier studies we visualized the telomerase RNA subunit (TR, U10569) by RNA fluorescence *in situ* hybridization (FISH) (Paeschke et al., 2008b; Postberg et al., 2005). These studies showed that TR is enriched in the replication band, a propagating accumulation of numerous replication foci, during S-phase. In this study immunofluorescence analyses using anti-TERT antibodies in combination with anti-StyRecQL antibodies (Figs. 3A,B,C) showed that, very similar to TR, the TERT subunit was enriched in the replication band. Furthermore, foci-like accumulations of TERT were observed within multiple spherical chromatin-poor nuclear bodies during S-phase outside of the replication band as well as in macronuclei during interphase suggesting discrete storage sites for nuclear proteins such as TERT and StyRecQL (Figs. 3A,B,C), similarly as proposed earlier for fibrillarin/Nop1p (Postberg et al., 2006). We assume that this difference between the two subunits of the telomerase complex — the existence of TERT storage sites even during interphase and, in contrast, the lack of TR detection outside of the replication band indicates that the functional telomerase complex becomes only assembled during S-phase in the replication band. Alternatively, but mutually not exclusively the comparably harsh treatment of samples prior to RNA FISH, which included denaturation steps to resolve secondary structures of TR, could have affected the morphology of sub-nuclear domains. Importantly, TERT and StyRecQL showed only marginal overlapping signals in spherical macronuclear bodies outside the replication band on the level of light microscopy using the *Colocalization Highlighter* module of ImageJ (Rasband, 1997–2004) software as described (Postberg et al., 2008). However, in the replication band almost all signals of both proteins overlapped as suggested by co-localization analyses (Figs. 3A,B). Here, co-localization of TERT and StyRecQL is illustrated by white voxels. To test whether a physical interaction between StyRecQL and TERT exists during replication we performed co-immunoprecipitation experiments either using telomerase extracts (Fig. 3D) or nuclear extracts (data not shown) from *Stylonychia* cells synchronized for S-phase enrichment as described earlier (Juranek et al., 2000). In both cases precipitation was done with the anti-StyRecQL antibody, since the chicken anti-TERT was not suited for immunoprecipitation experiments. Precipitated proteins were analyzed by Western analysis using either anti-StyRecQL or anti-TERT antibodies. As shown in Fig. 3D TERT (136.42 kDa) co-precipitates as a ~ 130–135 kDa protein using the anti-StyRecQL antibody from a telomerase extract demonstrating a physical association of both proteins. Similar results were obtained using macronuclear extracts for immunoprecipitation. In no case TERT co-precipitated with StyRecQL in extracts prepared from starved cells in which no replication bands can be detected (data not shown). Taking all these observations together we concluded that StyRecQL as well as TERT occurred within discrete foci/bodies within the chromatin-poor macronuclear compartment in the interphase nucleus and are enriched in the replication band. Furthermore our microscopical analyses as well as the co-immunoprecipitation experiments suggest that the TERT-TR-RecQ complex becomes assembled only during S-phase within the replication band and disassembles after replication.

**Fig. 4:**
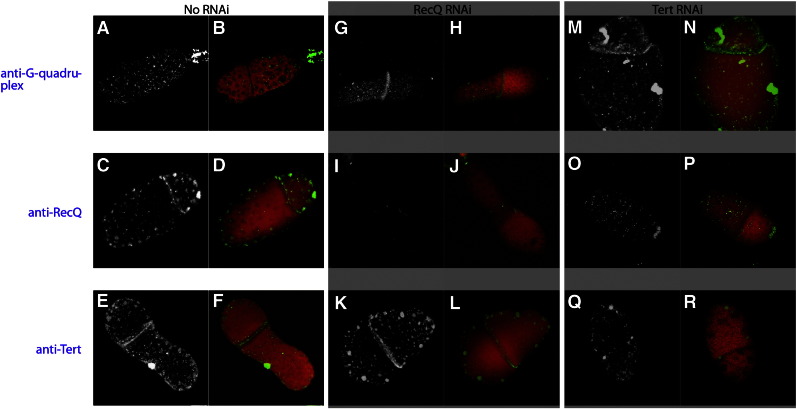
*Immunofluorescence analyses of RNAi experiments.* Immunofluorescence analyses of telomeric G-quadruplex DNA (A,B,G,H,M,N), StyRecQL (C,D,I,J,O,P) or TERT (E,F,K,L,Q,R) upon RNAi knock down of SyRecQ or, respectively, TERT expression. Image pairs are shown representing selectively the antigen of interest (gray: A,C,E,G,I,K,M,O,Q) and, respectively, a merged image (B,D,F,H,J,L,N,P,R) of the antigen of interest (green) with the counterstained DNA (red). All images are single light optical sections.

All these observations indicate that this telomerase-associated RecQ-like helicase may be involved in the replication of telomeres. To support this suggestion the expression of either the telomerase or the helicase (compare Fig. 1C, lane 2) was silenced by RNAi (Paeschke et al., 2005) and the effect on G-quadruplex structure and the relation between both proteins analyzed *in situ.* As in the case of silencing TERT by RNAi cells stop dividing after about 8 days of feeding bacteria, which express dsRNA directed against StyReyQL, almost no replication bands and morphological abnormal macronuclei are observed and cells eventually die after ten to twenty days of exposure to RNAi. The results of these silencing experiments are summarized in Fig. 4. In normal cells G-quadruplex DNA is organized within foci-like structures throughout the whole macronucleus, but no antibody staining can be detected in the replication band (Figs. 4A,B). This observation indicates that G-quadruplexes are resolved during replication as described before (Paeschke et al., 2005; Schaffitzel et al., 2001). In contrast, both, the telomerase as well as StyRecQL are highly enriched in the replication band (Figs. 4C–F). Upon silencing expression of StyRecQL no staining with the antibody against this protein can be detected in the macronucleus and the replication band demonstrating the efficiency of the silencing process (Figs. 4I,J). The distribution of telomerase was not affected (Figs. 4K,L) but importantly, G-quadruplex structures were still visible in the replication band showing that this helicase is required for resolving G-quadruplex structure during replication (Figs. 4G,H). When the expression of telomerase was silenced, G-quadruplex structures were still visible in the replication band (Figs. 4M,N) but also no staining with the anti-helicase antibody was observed in the replication band (Figs. 4O,P). In addition, the signal with the anti-G-quadruplex antibody seemed slightly weaker than in control cells probably due to the loss of telomeric sequences upon telomerase silencing. It can be concluded from these experiments that telomerase as well as a helicase is required for resolving G-quadruplex structure but that in this process probably the main function of telomerase is to recruit a telomerase associated helicase to the telomeres which in turn resolves G-quadruplex structure.

### 3.3 In vitro analyses

**Fig. 5:**
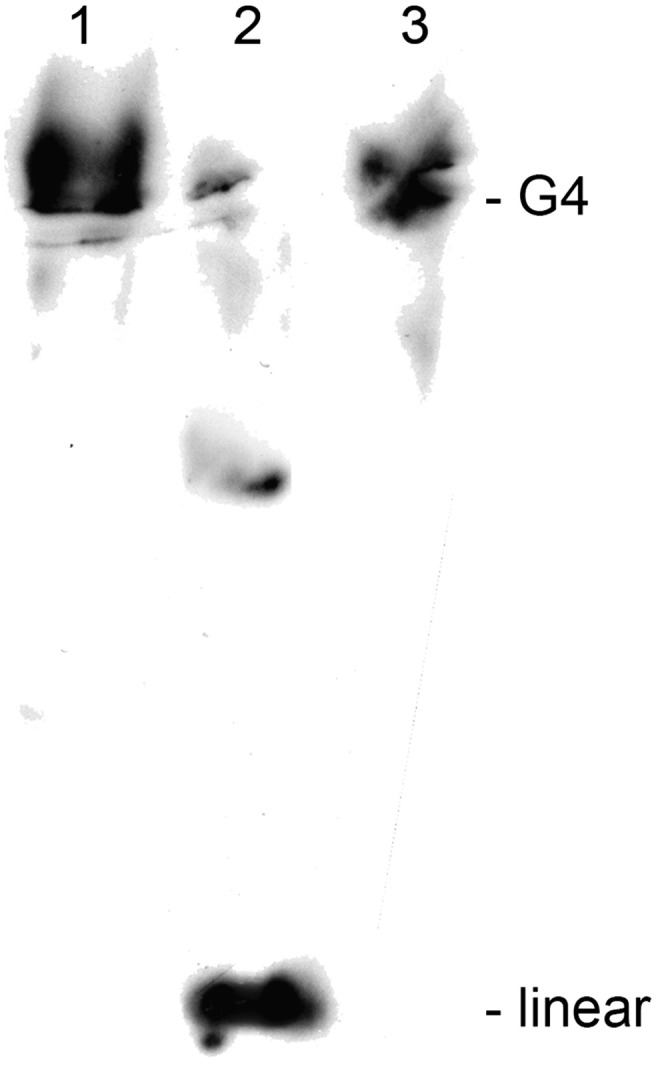
*In vitro telomerase assay.* Lane 1: High molecular weight telomeric G-quadruplex DNA. Lane 2: Linear telomeric DNA and a putative midsized intermediate conformation. Lane 3: G-quadruplex DNA is not resolved upon treatment with 1% SDS and heat (65 °C).

We made some preliminary experiments to detect a G-quadruplex resolving helicase activity in telomerase extracts. For this, we incubated the model telomere described before (Paeschke et al., 2005) consisting of the telomeric double stranded as well as the single strand 3′ overhang in 0.5 M NaCl overnight resulting in the formation of telomeric G-quadruplex DNA (Fig. 5, lane 1). Telomerase extract was added and incubated for various time intervals. This extract contains both the telomerase as well as StyRecQL as demonstrated by Western analyses. Resolution of G-quadruplex structure was analyzed by 10% PAGE (Sundquist and Klug, 1989). The results of these experiments did depend very much on the quality of the telomerase extract whose preparation cannot really be well controlled in these cells. However, in 3 independent experiments using 3 different telomerase extracts prepared from cells in S-phase we observed a shift from the high molecular weight G-quadruplex conformation (Fig. 5, lane 1) to the linear telomeric sequence (Fig. 5, lane 2). Such shifts never could be seen in extracts made from starved cells in which no replication is observed. In some cases an intermediate form was visible which may be the linear telomeric form to which a protein is bound (Fig. 5, lane 2). No shift towards the linear telomeric structure was observed when 1% SDS was added to the extracts and heated to 65 °C (Fig. 5, lane 3). These experiments indicate a G-quadruplex resolving helicase activity in nuclear extracts containing telomerase as well as StyRecQL. Due to the fact that cells in which telomerase or StyRecQL was silenced cannot be synchronized in S-phase and the low amount of cells available after these silencing experiments, we could not make the appropriate control extracts in which either telomerase or StyRecQL was silenced.

## 4 Discussion

There is increasing evidence that G-quadruplex structures may occur *in vivo* and may provide an elegant nucleic-acid based mechanism to modulate such important biological processes as replication, transcription or translation. A number of helicases have been shown to resolve G-quadruplex structures *in vitro* and mutations in some of these helicases have serious consequences for replication and genome stability *in vivo* (for review: (Lipps and Rhodes, 2009)). Until now, *in vivo* G-quadruplex formation at telomeres has only been demonstrated in ciliated protozoan and its regulation by the two telomere-end binding proteins, TEBPα and TEBPβ, throughout the cell cycle is well characterized (Paeschke et al., 2005, 2008b). It was shown that TEBPα binds to the 3′-rich G-overhang in a sequence specific manner, recruits TEBPβ to the ends (for review see (Price, 2006)) and the C-terminus of TEBPβ is required to fold the telomeric sequence into the G-quadruplex conformation (Paeschke et al., 2005). With the beginning of S-phase two phosphorylation sites in the C-terminus of TEBPβ become phosphorylated and this is a necessary prerequisite for G-quadruplex unfolding. However, the major function of phosphorylated TEBPβ is to recruit telomerase to the telomeres. Silencing telomerase expression also prevents G-quadruplex unfolding of G-quadruplex structure in the replication band (Paeschke et al., 2008b). However, neither phosphorylation of TEBPβ nor telomerase has been demonstrated to be sufficient to resolve telomeric G-quadruplex structure that allows telomere replication to proceed in a timely manner (Paeschke et al., 2008b). It therefore seemed to be reasonable to search for a helicase possibly involved in this process. From a *Stylonychia* macronuclear genomic database we identified sequence fragments encoding conserved helicase domains preferentially found in RecQ helicases.

Many DNA helicases are known to date and are considered to catalyze the unwinding of dsDNA in an energy-dependent manner. Particularly members of the RecQ family of helicases came into the focus of current research, because there is increasing evidence that these enzymes can unwind non-canonical DNA structures, such as G-quadruplex DNA, D-loops or Holliday junctions. RecQ helicases belong to the superfamily 2 of helicases and occur in bacterial and archaeal microbes as well as in eukaryotes. In humans defects in three of at least five functionally non-redundant genes encoding RecQ helicases are associated with severe heritable disease, i.e. Bloom's syndrome (BLM, RECQ2, RECQL3 gene), Rothmund-Thomson syndrome (RECQL4 gene) and Werner's syndrome (WRN, RECQ3, RECQL2 gene) (Bennett and Keck, 2004).

The complete 4.462 kb nanochromosome encoding a RecQ-like helicase (StyRecQL) was identified. The size of this helicase, which habours a central conserved helicase domain including several motifs homologous to known RecQ helicases of other eukaryotes, is 1152 aa (131.33 kDa). But unlike other RecQ helicase and similar to the RecQ-like protein 4 it lacks the RQC domain which harbors a Zinc finger motif involved in G4 binding. Antibodies were raised against peptides present in the conserved helicase domain. This antibody reacts with a single nuclear protein of about 130 KD and could be used for co-immunoprecipitation and *in situ* analyses. By *in situ* experiments as well as by silencing expression of either TERT or StyRecQL it clearly could be shown that this helicase is telomerase-associated in the replication band, recruited to replicating telomeres by telomerase and involved in G-quadruplex unfolding. It seems reasonable to assume that telomerase bound to phosphorylated TEBPβ recruits StyRecQL to the telomeres. This may explain that the RQC domain is not required in this helicase since it becomes specifically recruited to telomeres by telomerase. The factors involved in these processes still have to be characterized.

So far, we only could perform preliminary biochemical experiments to demonstrate the G-quadruplex resolving helicase activity *in vitro*. However, we could show that such a helicase activity is present in a nuclear extract in S-phase cells containing both telomerase as well as StyRecQL. This activity was not observed in starved cells but due to the technical problems related with RNAi treated cells as discussed above we could not make control extracts demonstrating that it is in fact StyRecQL which resolves telomeric G-quadruplex structure.

## 5 Conclusions

**Fig. 6:**
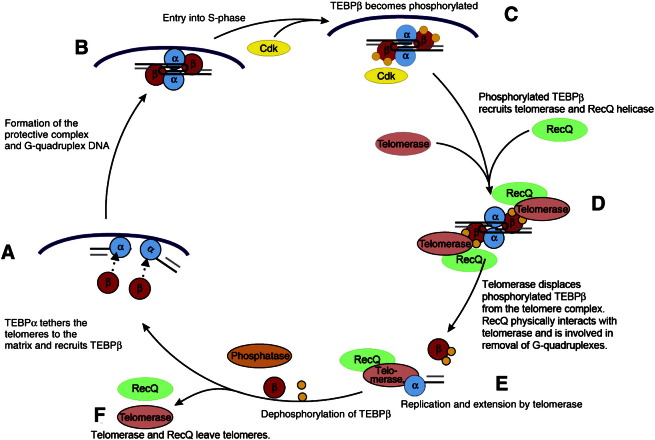
*A model for the regulation of telomeric G-quadruplex structure during the cell cycle.* In G1 and G2 phases TEBPalpha and TEBPbeta bind to the telomeres and promote their G-quadruplex formation. With the beginning of S-phase TEBPβ becomes phosphorylated. A complex composed of telomerase (TERT + TR) as well as RecQ-like helicase (StyRecQL) assembles within the replication band. Then this complex is recruited to the telomeres by phosphorylated TEBPβ forming an end-replication complex. Here, StyRecQL is involved in the rapid unfolding of G-quadruplex structure in replicating telomeres allowing the action of telomerase in telomere replication. At the end of S-phase TEBPβ becomes dephosphorylated. Subsequently the original telomere/TEBP complex becomes restored releasing the telomerase-RecQ complex from the telomere.

Nevertheless our observations now allow us to complete a mechanistic scheme for the regulation of telomeric G-quadruplex structure during the cell cycle (Fig. 6). In G1 and G2 phases TEBPα and TEBPβ bind to the telomeres and promote their G-quadruplex formation (Figs. 6A,B). With the beginning of S-phase TEBPβ becomes phosphorylated and recruits the telomerase-helicase complex, which becomes assembled in the replication band, to the end-replication machinery (Figs. 6C,D). The helicase is then involved in the rapid unfolding of G-quadruplex structure in replicating telomeres allowing the action of telomerase in telomere replication (Fig. 6E). At the end of S-phase TEBPβ becomes dephosphorylated restoring the original telomere/TEBP complex (Figs. 6F,A).

The following are the supplementary materials related to this article.

## Figures and Tables

**Table 1:** *Several conserved motifs characteristic for RecQ helicases are found in StyRecQL.* Consensus sequences of conserved motifs found in eukaryotic RecQ-like helicases and motifs present in *Stylonychia* StyRecQL.

	Consensus	StyRecQL
*motif 0*	Lxxx(F/Y/W)GxxxF(R/K)xxQ	LkerFGhetFKagQ
*motif 1*	TGxGKS	TGgGKS
*motif 2*	DExHC	DEiHC
*ssDNA recognition*	SxWGxxFRxxY	SeWshnFRpaY

## References

[bb0245] Ammermann D., Steinbruck G., Berger L.v., Hennig W. (1974). The development of the macronucleus in the ciliated protozoan *Stylonychia mytilus*. Chromosoma.

[bb0010] Baumann P., Cech T.R. (2001). Pot1, the putative telomere end-binding protein in fission yeast and humans. Science.

[bb0015] Bennett R.J., Keck J.L. (2004). Structure and function of RecQ DNA helicases. Crit. Rev. Biochem. Mol. Biol..

[bb0020] Bernstein D.A., Keck J.L. (2005). Conferring substrate specificity to DNA helicases: role of the RecQ HRDC domain. Structure.

[bb0025] Burge S., Parkinson G.N., Hazel P., Todd A.K., Neidle S. (2006). Quadruplex DNA: sequence, topology and structure. Nucleic Acids Res..

[bb0030] Curtis E.A., Landweber L.F. (1999). Evolution of gene scrambling in ciliate micronuclear genes. Ann. N. Y. Acad. Sci..

[bb0035] de Lange T. (2004). T-loops and the origin of telomeres. Nat. Rev. Mol. Cell Biol..

[bb0250] Dereeper A. (2008). Phylogeny.fr: robust phylogenetic analysis for the non-specialist. Nucleic Acids Res..

[bb0045] Edgar R.C. (2004). MUSCLE: multiple sequence alignment with high accuracy and high throughput. Nucleic Acids Res..

[bb0050] Felsenstein J. (1985). Confidence limits on phylogenies: an approach using the bootstrap. Evolution.

[bb0055] Froelich-Ammon S.J., Dickinson B.A., Bevilacqua J.M., Schultz S.C., Cech T.R. (1998). Modulation of telomerase activity by telomere DNA-binding proteins in Oxytricha. Genes Dev..

[bb0060] Gilson E., Geli V. (2007). How telomeres are replicated. Nat. Rev. Mol. Cell Biol..

[bb0065] Griffith J.D. (1999). Mammalian telomeres end in a large duplex loop. Cell.

[bb0255] Guindon S., Dufayard J.F., Lefort V., Anisimova M., Hordijk W., Gascuel O. (2010). New algorithms and methods to estimate maximum-likelihood phylogenies: assessing the performance of PhyML 3.0. Syst Biol.

[bb0075] Heyse K.S., Weber S.E., Lipps H.J. (2009). Histone modifications are specifically relocated during gene activation and nuclear differentiation. BMC Genomics.

[bb0080] Huber M.D., Duquette M.L., Shiels J.C., Maizels N. (2006). A conserved G4 DNA binding domain in RecQ family helicases. J. Mol. Biol..

[bb0085] Huber M.D., Lee D.C., Maizels N. (2002). G4 DNA unwinding by BLM and Sgs1p: substrate specificity and substrate-specific inhibition. Nucleic Acids Res..

[bb0090] Jones D.T., Taylor W.R., Thornton J.M. (1992). The rapid generation of mutation data matrices from protein sequences. Comput. Appl. Biosci..

[bb0095] Juranek S., Jönsson F., Maercker C., Lipps H.J. (2000). The telomeres of replicating macronuclear DNA molecules of the ciliate *Stylonychia lemnae*. Protistology.

[bb0100] Linger B.R., Price C.M. (2009). Conservation of telomere protein complexes: shuffling through evolution. Crit. Rev. Biochem. Mol. Biol..

[bb0105] Lipps H.J., Rhodes D. (2009). G-quadruplex structures: *in vivo* evidence and function. Trends Cell Biol..

[bb0110] Liu Z. (1999). The three-dimensional structure of the HRDC domain and implications for the Werner and Bloom syndrome proteins. Structure.

[bb0115] Maizels N. (2006). Dynamic roles for G4 DNA in the biology of eukaryotic cells. Nat. Struct. Mol. Biol..

[bb0120] Oganesian L., Moon I.K., Bryan T.M., Jarstfer M.B. (2006). Extension of G-quadruplex DNA by ciliate telomerase. EMBO J..

[bb0125] Paeschke K., Juranek S., Rhodes D., Lipps H.J. (2008). Cell cycle-dependent regulation of telomere tethering in the nucleus. Chromosome Res..

[bb0130] Paeschke K., Juranek S., Simonsson T., Hempel A., Rhodes D., Lipps H.J. (2008). Telomerase recruitment by the telomere end binding protein-beta facilitates G-quadruplex DNA unfolding in ciliates. Nat. Struct. Mol. Biol..

[bb0135] Paeschke K., McDonald K.R., Zakian V.A. (2010). Telomeres: structures in need of unwinding. FEBS Lett..

[bb0140] Paeschke K., Simonsson T., Postberg J., Rhodes D., Lipps H.J. (2005). Telomere end-binding proteins control the formation of G-quadruplex DNA structures *in vivo*. Nat. Struct. Mol. Biol..

[bb0145] Parfrey L.W., Lahr D.J., Knoll A.H., Katz L.A. (2011). Estimating the timing of early eukaryotic diversification with multigene molecular clocks. Proc. Natl. Acad. Sci. U. S. A..

[bb0150] Patel D.J., Phan A.T., Kuryavyi V. (2007). Human telomere, oncogenic promoter and 5′-UTR G-quadruplexes: diverse higher order DNA and RNA targets for cancer therapeutics. Nucleic Acids Res..

[bb0155] Petersen B., Petersen T.N., Andersen P., Nielsen M., Lundegaard C. (2009). A generic method for assignment of reliability scores applied to solvent accessibility predictions. BMC Struct. Biol..

[bb0160] Postberg J., Alexandrova O., Cremer T., Lipps H.J. (2005). Exploiting nuclear duality of ciliates to analyse topological requirements for DNA replication and transcription. J. Cell Sci..

[bb0165] Postberg J., Alexandrova O., Lipps H.J. (2006). Synthesis of pre-rRNA and mRNA is directed to a chromatin-poor compartment in the macronucleus of the spirotrichous ciliate *Stylonychia lemnae*. Chromosome Res..

[bb0170] Postberg J., Heyse K., Cremer M., Cremer T., Lipps H.J. (2008). Spatial and temporal plasticity of chromatin during programmed DNA-reorganization in Stylonychia macronuclear development. Epigenetics Chromatin.

[bb0175] Price C., Lange T.d., Lundblad V., Blackburn E. (2006). Ciliate Telomeres. Telomeres.

[bb0260] Rasband W.S. (1997–2004). ImageJ.

[bb0185] Rhodes D., Giraldo R. (1995). Telomere structure and function. Curr. Opin. Struct. Biol..

[bb0190] Saitou N., Nei M. (1987). The neighbor-joining method: a new method for reconstructing phylogenetic trees. Mol. Biol. Evol..

[bb0195] Schaffitzel C., Berger I., Postberg J., Hanes J., Lipps H.J., Pluckthun A. (2001). In vitro generated antibodies specific for telomeric guanine-quadruplex DNA react with *Stylonychia lemnae* macronuclei. Proc. Natl. Acad. Sci. U. S. A..

[bb0200] Schaffitzel C., Postberg J., Paeschke K., Lipps H.J. (2010). Probing telomeric G-quadruplex DNA structures in cells with in vitro generated single-chain antibody fragments. Methods Mol. Biol..

[bb0205] Sigrist C.J. (2010). PROSITE, a protein domain database for functional characterization and annotation. Nucleic Acids Res..

[bb0210] Sundquist W.I., Klug A. (1989). Telomeric DNA dimerizes by formation of guanine tetrads between hairpin loops. Nature.

[bb0265] Tamura K., Dudley J., Nei M., Kumar S. (2007). MEGA4: Molecular Evolutionary Genetics Analysis (MEGA) software version 4.0. Mol. Biol. Evol..

[bb0220] Vega L.R., Mateyak M.K., Zakian V.A. (2003). Getting to the end: telomerase access in yeast and humans. Nat. Rev. Mol. Cell Biol..

[bb0225] Wang F. (2007). The POT1–TPP1 telomere complex is a telomerase processivity factor. Nature.

[bb0270] Wang Q. (2011). G-quadruplex formation at the 3′ end of telomere DNA inhibits its extension by telomerase, polymerase and unwinding by helicase. Nucleic Acids Res..

[bb0235] Zahler A.M., Williamson J.R., Cech T.R., Prescott D.M. (1991). Inhibition of telomerase by G-quartet DNA structures. Nature.

[bb0240] Zhang M.L. (2010). Yeast telomerase subunit Est1p has guanine quadruplex-promoting activity that is required for telomere elongation. Nat. Struct. Mol. Biol..

